# Investigating psychological and motivational predictors of problematic smartphone use among Smartphone-based Social Networking Service (SNS) users

**DOI:** 10.1016/j.abrep.2023.100506

**Published:** 2023-06-21

**Authors:** Min-Jung Kwak, Dai-Jin Kim

**Affiliations:** a Department of Counseling and Art Therapy, Graduate School of Education, Hongik University, Seoul, Republic of Korea; b Department of Psychiatry, College of Medicine, Seoul St. Mary’s Hospital, The Catholic University of Korea, Seoul, Republic of Korea

**Keywords:** Anxiety, Logistic regression, Problematic smartphone use, Reward responsiveness, Self-control, Smartphone-based SNS users

## Abstract

Given that the active use of certain smartphone applications is associate with problematic smartphone use, it has been proposed that certain smartphone applications are more addictive than others, such as Social Networking Services (SNS). Still, studies that consider smartphone users’ main usage application which are known to influence the users’ problematic smartphone use, such as SNS, remain to be explored. Thus, the current study aims to investigate the psychological and motivational predictors of problematic smartphone use in a sample of smartphone-based SNS users whose main device usage is SNS. A series of mean comparison tests and binary logistic regression were performed in this study. Of the 433 smartphone-based SNS users, 218 were male (50.3%) and 215 were female (49.7%). Age of 433 participants ranged from 20 to 40, and mean age was 30.75 (SD = 7.84). 73 participants (16.9%) were sorted into the high-risk problematic smartphone use group and 360 participants (83.1%) were categorized as the normal user group. The finding from binary regression analysis showed that reward responsiveness from the Behavioral Activation System (BAS), a lack of self-control, and anxiety significantly increased the odds of problematic smartphone use of the smartphone-based SNS users. Reward responsiveness was found to be the most powerful predictor. Our findings broaden the existing literature and provide implications to reduce addictive smartphone use relating to smartphone-based SNS usage.

## Highlights

• Social Networking Service (SNS) application has been suggested as an influential application that cause problematic smartphone use.
• Exploring the psychological and motivational predictors of problematic smartphone use among smartphone-based SNS users.
• Reward responsiveness is the most potent predictor on problematic smartphone use.
• Low self-control is a predictor to increase the risk of problematic smartphone use.
• Anxiety predicts the higher risk of problematic smartphone use.

## 1 Introduction

In modern society, smartphones are essential technology that allow users to access various contents regardless of time and place (Jung, Kim, & Chan-Olmsted, 2014). In conjunction with the rapid growth of smartphone users, a issue of the problematic smartphone use has emerged as the overuse of smartphones results in adverse effects on the physical, psychological, social, or financial aspects of users’ lives (Kim et al., 2016). Though problematic smartphone use has regarded as an addictive behavior (Hong, Yeom, & Lim, 2021), it might be inappropriate to apply the word “addiction” to problematic smartphone usage because an official clinical diagnosis has not yet existed in the 5th Edition of Diagnostic and Statistical Manual of Mental Disorders (DSM-5) (Kim et al., 2016). Thus, the current study will use ‘problematic smartphone use’ to indicate the addictive usage of smartphones.

Problematic smartphone use is a maladaptive dependency on smartphone usage which can cause a failure to control one’s urge to use one’s smartphone despite negative consequences (Kwak, Cho, & Kim, 2022). Although the number of smartphone users who addictively use their devices has been dramatically increasing (Griffiths, 2010), it is essential to acknowledge that not all smartphone users display problematic device usage. Indeed, Kuss and Griffiths (2011) proposed that smartphone users do not become addicted to the device itself but to the contents they actively use. Previous findings also inclined that some smartphone application contents are more likely to cause problematic smartphone use than others because the most-used application contents of smartphones may decide the usage pattern of smartphone users (Griffiths, 2010, Jeong et al., 2016, Kwak et al., 2022). Especially, Social Networking Service (SNS) has been suggested as influential content highly related to problematic smartphone use (Jeong et al., 2016, Park and Hwang, 2014, Turel and Serenko, 2012; Van Deursen, Bolle, Hegner, & Kommers, 2015). SNS is a web-based platform such as Facebook, Instagram, or Twitter that provides not only social but also emotional, entertaining, and informational content (Jeong et al., 2016, Kwak et al., 2022). With the advent of smartphones, numerous smartphone users use SNS applications regardless of time and space via their devices (Silver and Huang, 2019, Turel and Serenko, 2012). The frequent use of SNS applications via smartphone devices can increase problematic smartphone use. Findings from previous studies suggested that SNS is the most influential application, which is linked to a higher risk of problematic smartphone use (Jeong et al., 2016). Also, the frequent usage of SNS applications was associated with problematic smartphone use (e.g., Kwak et al., 2022, Lee et al., 2020, Salehan and Negahban, 2013).

However, not all individuals whose primary smartphone usage is SNS, who will be defined as ‘smartphone-based SNS users’ in this study, would problematically use their devices (Griffiths, 2013). Hence, it is necessary to explore which psychological and motivational factors of smartphone-based SNS users would predict their problematic smartphone use. Even though there have been numerous studies that investigated psychological or motivational predictors on problematic smartphone use, the participants of the existing studies were ‘general users’ whose primary smartphone usage content has not been identified (e.g., Barnes et al., 2019, Koç and Turan, 2021). In addition, although there have been a few studies that targeted smartphone-based SNS users (e.g., Kwak et al., 2022; Luqman, Masood, Shahzad, Shahbaz, & Feng, 2021), those research did not explore which predictors may increase the odds of problematic smartphone use. Instead, the findings of previous studies on smartphone-based SNS use suggested the adverse effect of late-night usage of smartphone-based SNS use (Luqman et al., 2021) or the effect of direct and indirect paths of variables contributing to problematic smartphone use (Kwak et al., 2022). Thus, a study that examines predictors of problematic smartphone use within the context of smartphone-based SNS should be conducted to explore which psychological and motivational variables would be associated with problematic smartphone use more accurately while considering main smartphone usage content. Therefore, the current study aims to investigate psychological and motivational predictors of problematic smartphone use in the sample of smartphone-based SNS users. The present research will offer a clearer understanding of the nature of problematic smartphone use than the existing literature. It will clarify a more precise relationship between psychological and motivational variables and problematic smartphone use while considering the use of SNS on problematic smartphone use.

A growing body of literature has emphasized several psychological and motivational variables such as anxiety, depression, low self-control, Behavioral Inhibition System (BIS), and the Behavioral Activation System (BAS), as those variables have been known to influence both problematic smartphone and SNS use. Anxiety and depression are known to cause adverse effects on psychological and physical health and harm the quality of life (Geng, Gu, Wang, & Zhang, 2021). Previous research suggested that an individual with high anxiety and/or depression tends to maladaptively indulge in specific behavior to alleviate those negative psychological symptoms (Kim, Seo, & David, 2015; Lepp, Barkley, & Karpinski, 2014). Some studies reported a significant association between a higher risk of problematic smartphone use and a higher level of anxiety (Hong et al., 2012, Hussain et al., 2017; Park & Choi, 2015) and depression (Alhassan et al., 2018, Elhai et al., 2019, Yuan et al., 2020). Previous studies also indicated that problematic SNS use had been associated with a high level of anxiety and/or depression (Andreassen et al., 2016, Kuss and Griffiths, 2017, Shensa et al., 2017, Thorisdottir et al., 2019). Self-control refers to the ability to control impulses by monitoring one’s automatic behaviors, thoughts, and emotions (Sok, Seong, & Ryu, 2019). The primary function of self-control is resisting temptations and inhibiting undesirable behaviors to achieve one’s goal (Tang, Posner, Rothbart, & Volkow, 2015). Thus, the failure of self-control is related to various types of addictive behaviors, including problematic use of smartphones (Cho, Kim, & Park, 2017; Jiang & Zhao, 2016; Servidio, 2019; Sok, Seong, & Ryu, 2019) and SNS (Kuss and Griffiths, 2017, Salehan and Negahban, 2013, van Deursen and J. A. M., Bolle, C. L., Hegner, S. M., & Kommers, P. A. M. , 2015; Van Endert & Mohr, 2020). The BIS and BAS are the neuropsychological motivation systems that impact on one’s motivation, affect, and behavior (Carver & White, 1994). The BIS controls inhibitory behaviors in response to adverse effects, such as punishments (Carver & White, 1994). The BAS activates behaviors in response to positive outcomes, such as rewards. (Carver & White, 1994). The BAS consists of BAS_drive (the persistent pursuit of desired goals); BAS_fun seeking (a desire for novel rewards and a willingness to approach pleasurable events); and BAS_reward responsiveness (positive responses to the occurrence of rewards) (Carver & White, 1994). In line with Nam and colleagues’ (2018) suggestion that high engagement in behaviors can vary depending on the level of an individual’s motivation towards reward or punishment, findings from a growing body of literature proposed that the high level of BIS and/or BAS sensitivity predispose to addictive behavior (Franken, Muris, & Georgieva, 2006), including problematic smartphone (Kim et al., 2016, Kwak et al., 2022) and SNS use (Abbasi et al., 2016, Nam et al., 2018).

Therefore, the current study will investigate the predicting power of anxiety, depression, low self-control, and BIS/BAS on problematic smartphone use in a sample of smartphone-based SNS users. Those variables will be considered potential predictors that may significantly predict the problematic smartphone use of smartphone-based SNS users.

## 2 Materials and methods

### 2.1 Participants

From 5,003 survey respondents collected by a professional polling company (Hankook Research, Inc, Seoul, Korea), 433 participants who reported their primary smartphone usage as SNS were selected after ruling out the sample with psychiatric disorders and treating a missing value. The 433 participants are defined as “smartphone-based SNS users” in this study. All participants were aged from 20 to 49 and resided in a metropolitan area in South Korea. Informed consent was obtained online from all survey respondents before participation, and those who refused to consent were excluded.

Demographic information, average smartphone usage hours, the Korean Version of the Smartphone Addiction Proneness Scale for Adults (K-SAPS,) depression and anxiety subscale from the Symptom Checklist-90-Revised (SCL-90-R), Brief Self-Control Scale (BSCS), Behavioral Inhibition System (BIS), and Behavioral Activation System (BAS), were administered.

### 2.2 Measurements

#### 2.2.1 A Korean version of smartphone addiction Proneness scale (K-SAPS).

The K-SAPS for adults (National Information Society Agency, 2011) measured problematic smartphone use status. The scale contains four subdomains: (1) disturbance of adaptive functions; (2) virtual life orientation; (3) withdrawal; and (4) tolerance. The K-SAPS consists of 15 Likert-type items (1 = *Strongly disagree* to 4 = *Strongly agree*) and contains items such as “There have been times when I was unable to concentrate on my work/study due to smartphone use.” In this sample, Cronbach’s alpha was 0.903. Among the 433 users, 73 participants (16.9%) were sorted into the high-risk problematic smartphone use (HPSU) group, and 360 participants (83.1%) were categorized as the normal user (NU) group, using the K-SAPS cutoff score for the risk of problematic smartphone use, which is 40.

#### 2.2.2 The Brief Self-Control Scale.

The current study used the Korean version of the Brief Self-Control Scale (BSCS) (Hong, Kim, Kim, & Kim, 2012) in order to measure a low level of self-control. The BSCS is a 13-item, a 5-point Likert scale (1 = *Not at all* to 5 = *Very much*) to assess one’s ability to control thoughts, emotions, performance regulation, habit-breaking, and impulsivity (Hong et al., 2012). It contains items such as “Pleasure and fun sometimes keep me from getting work done.” Higher scores on this scale represent lower levels of self-control. In this sample, Cronbach’s alpha was 0.724.

#### 2.2.3 Depression and anxiety scales from the Symptom Checklist-90-Revised (SCL-90-R).

The depression and anxiety subscales from the Korean version of SCL-90-R (Kim, Won, & Kim, 2016) were used. The 13 items for depression were rated on a 5-point Likert scale (0 = *Not at all* to 5 = *Extremely*). The scale contains items such as “I feel lonely,” indicating the severity of participants’ depression symptoms in the past week. In this sample, Cronbach’s alpha was 0.942. The ten items for anxiety were rated on a 5-point Likert scale (0 = *Not at all* to 5 = *Extremely*), indicating the severity of participants’ anxiety symptoms in the past week. It includes items such as “I feel nervous.” In this sample, Cronbach’s alpha was 0.944.

#### 2.2.4 Behavioral inhibition system (BIS) and behavioral activation system (BAS) scales**.**

The Korean version of BIS and BAS scales (Kim & Kim, 2001) were used to measure BIS/BAS sensitivity. The BIS is a 7-item self-report questionnaire designed to assess aversive motivation related to suppressing behavior towards aims. Items were rated on a 4-point Likert scale (1 = *Strongly disagree* to 4 = *Strongly agree*). In this sample, Cronbach’s alpha was 0.78. It contains items such as “I feel pretty worried or upset when I think or know somebody is angry at me.” The BAS is a 13-item self-report questionnaire designed to assess the motivational activation of behavior towards goals in three domains: reward responsiveness (5 items, “When good things happen to me, it affects me strongly”), drive (4 items, “When I go after something, I use a ‘no holds bared’ approach”), and fun seeking (4 items, “I crave excitement and new situations”). Items were rated on a 4-point Likert scale (1 = *Strongly disagree* to 4 = *Strongly agree*). In this sample, Cronbach’s alphas were 0.844, 0.769, and 0.772 for reward responsiveness, drive, and fun seeking, respectively. Each BAS subscale was included in the statistical analysis as a subcomponent to examine the unique contribution of the subscales.

## 3 Statistical analysis

All statistical analyses were conducted using SPSS/WIN 21.0 (IBM, Armonk, NY, USA) and AMOS 23.0 (SPSS, Chicago, IL, USA). Descriptive statistics included frequency, mean, standard deviation, and percentage, which were used to describe participants’ sociodemographic characteristics, daily hours of using smartphones and SNS applications, and study variables. Skewness and kurtosis were examined to confirm that study variables were normally distributed. A series of independent sample t-tests and chi-square tests were used to detect whether there would be significant differences in demographic variables and either smartphone or SNS usage patterns between the HPSU and NU groups. To examine the unique contribution of psychological and motivational variables that were significant predictors, binary logistic regression analyses of problematic smartphone use status were used.

## 4 Results

### 4.1 Sample demographics of participants

The number of smartphone-based SNS users was 433. Of the users, 215 were female (49.7%), and 218 were male (50.3%). Participants were at least 20 (mean age = 30.75; *SD* = 7.84). The distribution of the age groups is as follows: 20–29 years (49.9%), 30–39 years (33.3%), and 40–49 years (16.9%). Participants had achieved approximately two years of education in addition to a high school diploma or equivalent (*M* = 2.62, *SD* = 0.50). Most participants were employed (part or full-time) or enrolled as students (*n* = 356, 82.2%). Over half of the participants (*n* = 267, 61.7%) were single, and fewer than one in four were married (*n* = 154, 35.6%). The remaining participants were divorced, separated, or in a domestic partnership. Regarding the history of using smartphones, 425 users (98.2%) reported that they have been using smartphones for at least two years. Regarding average use per day, participants spent about 54% of their time on SNS, while the average time of SNS use was seven hours weekly.

### 4.2 Chi-square test for demographic variables by the risk of problematic smartphone use status

Demographic variables such as age, sex, and race are common confounders, as suggested by descriptive epidemiology (Wacholder, McLaughlin, Silverman, & Mandel, 1992). Therefore, we conducted a Chi-square test to compare sex, age, level of education, job status, relationship/marriage status, and economic status between NU and HPSU and find a confounder. The difference in the demographic information according to the risk of problematic smartphone use status is presented in Table 1. The chi-square test results showed that there was a significant sex difference (χ^2^ = 5.01, *p* <.05) and education level (χ^2^ = 6.26, *p* <.05) according to the two groups. All other bivariate tests for age, job, marital status, and wealth quintile according to problematic smartphone use did not predict the risk of problematic smartphone use. Hence, sex and education level will be included as control variables in block 1 of the logistic regression model.

**Table 1:** Chi-square test for demographic variables by the group status.

	Problematic usage of smartphone status		
	NU (%n)	HPSU (%n)	*χ^2^*
**Sex**			
Male	43.9(1 9 0)	6.5(28)	5.05*
Female	39.3(1 7 0)	10.4(45)	
**Age**			
20–29	40.9(1 7 7)	9.0(39)	0.75
30–39	27.7(1 2 0)	5.5(24)	
40–49	14.5(63)	2.3(10)	
**Level of education**			
Below middle school graduated	0.2(1)	0.5(2)	6.26*
High school graduated	30.0(1 3 0)	6.9(30)	
Above university/college graduated	52.0(2 2 9)	9.5(41)	
**Job status**			
Student	18.9(82)	4.6(20)	17.72
Employee	33.6(1 6 8)	6.8(34)	
Professional	9.5(41)	2.3(10)	
No job	2.5(11)	0.9(4)	
Others	11.6(58)	1(5)	
**Relationship/Marriage status**			
Not married	51.3(2 2 2)	10.4(45)	5.63
Living with a partner	0.7(3)	0.5(2)	
Married	30.3(1 3 1)	5.3(23)	
Divorced/Separated	0.9(4)	0.7(3)	
**Economic states**			
Lowest	9.9(43)	2.3(10)	1.24
Second	29.1(1 2 6)	5.1(22)	
Middle	34.4(1 4 9)	7.4(32)	
Fourth	9.2(40)	1.8(8)	
Highest	0.5(2)	0.2(1)	
*Note.* NU = normal user group; HPSU = high-risk of problematic smartphone use group; (%n) = percent (number). *p <.05.			

### 4.3 Mean differences of smartphone and SNS usage time by the group status

In the present study, two sample *t*-test was conducted to examine the mean differences in smartphone and SNS application usage time by group status (Table 2). The result indicated significant differences in the usage patterns for smartphones and SNS, depending on the risk of problematic smartphone use. Compared to individuals in the NU group, those in the HPSU group reported a significantly longer smartphone usage time on weekdays and weekends. The HPSU group also engaged in more prolonged SNS application use after starting smartphone use than the NU group. Moreover, the HPSU group reported longer involvement in SNS applications on weekdays and weekends.

**Table 2:** Mean differences of smartphone and SNS application usage time by the group status.

	NU (*n* = 360)			HPSU (*n* = 73)	*t-*test
	M	(SD)	M	(SD)	
The daily average usage time of smartphone (on weekdays)	4.71	3.77	6.93	4.56	−3.90^***^
The daily average usage time of smartphone (on weekends)	4.94	3.83	7.40	4.56	−4.23^***^
SNS usage time after once started by using smartphone	1.76	1.46	2.34	1.55	−3.07^**^
The time of using SNS applications (on weekdays)	2.03	1.91	3.15	2.36	−3.81^***^
The time of using SNS applications (on weekends)	2.17	1.80	3.45	3.23	−3.28^**^
*Note.* NU = normal user group; HPSU = high-risk of problematic smartphone use group. ^**^p <.01; ^***^p <.001.					

### 4.4 The predictors of the problematic smartphone use of Smartphone-based SNS users

The binary logistic regression analysis results can be seen in Table 3. We used the risk of problematic smartphone use status (1 = HPSU; high risk of problematic smartphone use, 0 = NU; normal users) as the dependent variable. The independent variables were self-control, depression, and anxiety subscales from the SCL-90-R, BIS, and BAS.

**Table 3:** Binary logistic regression analysis for predicting problematic smartphone use of smartphone-based SNS users (n = 433).

Variables						
		S.E	Wald	Sig.	OR	95% CI for OR
**Block 1**						
Sex	-0.599	0.264	5.514	0.023	0.55	0.33–0.92
Level of education	-0.402	0.251	2.562	0.109	0.67	0.41–1.10
						
**Block 2**						
BSCS	0.073	0.030	5.740	0.017	1.08	1.02–1.14
Depression	-0.009	0.034	0.076	0.783	0.99	0.93–1.06
Anxiety	0.117	0.045	6.846	0.009	1.13	1.03–1.23
BIS	0.011	0.073	0.022	0.882	1.01	0.88–1.17
BAS						
Reward Responsiveness	0.312	0.116	7.222	0.007	1.37	1.10–1.72
Drive	0.062	0.110	0.316	0.574	1.06	0.86–1.32
Fun seeking	-0.035	0.107	0.107	0.744	0.97	0.78–1.19
*Note.* BSCS = Brief Self-Control Scale; BIS = Behavioral inhibition system; BAS = Behavioral Approach System.						

A two-block binary logistic regression analysis was conducted. In order to examine the unique contribution of the variables, sex, and education level were included as control variables in Block 1 since those were significant variables for the risk of problematic smartphone use in our sample. In addition to these two demographic variables, self-control, depression, anxiety, BIS, and the factors from the BAS were included in Block 2. The first block of the model correctly classified approximately 83% of cases. The demographic variables explained 20% of the variation in data (Nagelkerke *R^2^*) and demonstrated an adequate chi-square goodness-of-fit (χ^2^(1) = 5.086, *p* < 05). According to the binary logistic regression analysis, gender (*p* <.05) was a significant predictor when entered as a control variable in Block 1, indicating that females had a greater risk of problematic smartphone use. However, gender became a non-significant variable when the other variables were entered in Block 2. The second block of the model correctly classified approximately 86% of cases, explained 39% of the variation in data (Nagelkerke *R^2^*), and demonstrated an adequate chi-square goodness-of-fit (χ^2^ (8) = 115.017, *p* 〈0 0 1).

Among the variables, the BSCS was a significant predictor of problematic smartphone use (*OR* = 1.08, 95% *CI* = 1.02–1.14, *p* <.05). Our result indicated that each increment of the BSCS would raise the chance of problematic smartphone use by 8%. Anxiety also significantly predicted problematic smartphone use (*OR* = 1.13, 95% *CI* = 1.03–1.23, *p* <.01). Increments at each anxiety level increased the possibility of problematic smartphone use by 13%. Additionally, those with a higher BAS score for reward responsiveness had a higher probability of potentially engaging in problematic smartphone use (*OR* = 1.37, 95% *CI* = 1.10–1.72, *p* <.01), indicating that each point increment increases in the reward responsiveness level resulted in an approximately 37% greater likelihood of problematic smartphone use. This result showed that reward responsiveness was the most potent predictor of problematic smartphone use in our sample of smartphone-based SNS users. Depression, the BIS variables, and the BAS drive and fun seeking did not predict problematic smartphone use in smartphone-based SNS users.

## 5 Discussion

The present study identified the psychological predictors of problematic smartphone use in the sample of smartphone-based SNS users. To our best knowledge, it is the 1st study that explored the predictors of problematic smartphone use considering the main application content that participants use. In our study, self-control, anxiety, and BAS reward responsiveness played a crucial role in predicting the risk of smartphone-based SNS users’ problematic smartphone use. Hence, the results of the current study will broaden the existing literature while considering the influence of SNS application usage on problematic smartphone use.

In this study, reward responsiveness from the BAS was the most influential predictor of problematic smartphone use in our sample. That is, smartphone-based SNS users with greater reward dependency tend to engage in problematic smartphone use more. The result was similar to those of prior studies that suggested high reward sensitivity is associated with problematic media use (Franken et al., 2006, Nam et al., 2018). Previous findings indicated that SNS users tend to be guided by the extrinsic/intrinsic rewarding experience from their SNS activity, such as enjoyment, satisfaction, or self-enhancement (Kim et al., 2011, Shibchurn and Yan, 2015), which stimulates users’ reward sensitivity (Burgess, 2015). As the high level of reward sensitivity of the users makes it difficult for them to control their cravings to gain immediate rewards (Abbasi et al., 2016, Balconi et al., 2014), Such experiences from SNS activity may let SNS users pursue immediate gratification and follow their impulses to get more rewards from their SNS usage. In turn, smartphone-based SNS users may be prone to excessively using SNS applications via their smartphones to get more pleasurable outcomes, which causes problematic smartphone use. In addition, SNS applications have a reward-granting system, such as an instant notification that functions as a reward (Jeong et al., 2016). This functional feature stimulates SNS users’ reward-responsiveness tendency by making the users expect satisfaction immediately following the instant notification (Park & Hwang, 2014; Van Deursen et al., 2017; Wilmer & Chein, 2016). Although it would not cause problematic behavior to the technology itself but to the positive feelings that ‘likes’ and positive comments of appreciation can produce (Kuss & Griffiths, 2017), such a notification system may increase smartphone-based SNS users’ reward sensitivity and ultimately accelerate their addictive smartphone checking behavior. Therefore, the users could develop strategies to decrease their urges to seek these rewards. Thus, clinical interventions for smartphone-based SNS users require a thorough understanding of the users’ behavioral tendency to seek rewards, influencing the temptation to check their smartphones.

Low self-control also significantly enhanced the risk of problematic smartphone use in the sample of smartphone-based SNS users. The result implies that the participants with low self-control capacity tend to engage in problematic smartphone use. Our finding aligns with previous works that proposed a lack of self-control increased the risk of problematic smartphone use (e.g., Berger et al., 2018, Cho et al., 2017, Kwak et al., 2022). SNS applications provide instant notifications relating to users’ SNS activities which provide immediate pleasure only with a slight touch on the screen (Hong et al., 2021). Such a system may lead smartphone-based SNS users with low self-control to fail to manage their urges to use their devices against tempting SNS application signals. In turn, the users may be unable to control their intense craving to immediately respond to the received notifications that cause continuous usage of SNS applications. Such media usage patterns will ultimately increase the risk of problematic smartphone use. Therefore, it would be essential for smartphone-based SNS users to enhance their self-control capacity to prevent problematic smartphone use. Clinicians specializing in problematic smartphone use should attempt to devise practical interventions that enable smartphone-based SNS users to regulate their cravings for device usage.

In line with previous studies that emphasized a predicting role of anxiety in problematic smartphone use (e.g., Elhai et al., 2017, Hawi and Samaha, 2017, Rozgonjuk et al., 2018), anxiety was a significant predictor of the risk of problematic smartphone use among smartphone-based SNS users. The finding indicates that smartphone-based SNS users with high anxiety levels are prone to the addictive use of smartphones. Individuals with high anxiety levels tend to pursue social reassurance by constantly checking others’ reactions and connecting with others (Assunção & Matos, 2017; Cougle et al., 2012). As one of the functions of SNS is fulfilling one’s social needs (Kuss and Griffiths, 2017, Oberst et al., 2017), the participants with high anxiety those who possess reassurance-seeking tendency may habitually check their SNS applications to stay up to date, connect with others, and thereby alleviate their anxiety. Given that our study sample consisted of those who primarily use their smartphones for SNS, anxiety might lead smartphone-based SNS users to habitually use SNS applications via their smartphones to calm their anxiety by gaining social reassurance from others. Such behavior may increase the odds of problematic smartphone use. Therefore, reducing anxiety related to relational assurance may be needed for smartphone-based SNS users to lower their addictive smartphone usage. By monitoring smartphone-based SNS users’ anxiety-reducing motives for using their smartphones, clinicians could investigate how their smartphone usage pattern is associated with their internal disturbance and propose more constructive smartphone usage habits for the users.

Inconsistent with findings from existing literature (e.g., Alhassan et al., 2018, Kim et al., 2016), depression, the BIS, drive, and fun-seeking of the BAS did not predict the risk of problematic smartphone use of smartphone-based SNS users. One of the main functions of SNS applications is to promote social-oriented behaviors (Kuss and Griffiths, 2017, Kwak et al., 2022). Given that our sample consisted of smartphone users whose primary purpose for using smartphones is SNS, the smartphone habits of our participants may be driven more by social-oriented motives, such as anxiety rather than depression. Also, perhaps the BIS and other subcomponents of BAS play a less dominant role in predicting smartphone-based SNS users’ addictive device usage despite their potential impact on problematic smartphone use. Future research should navigate more evident role of those variables on problematic smartphone use.

There are a few limitations that need to be addressed. First, the cross-section design of this study could not provide confirmed causation between predictors of problematic smartphone use. It is recommended that future studies to investigate a more evident cause-effect relationship between the variables and problematic smartphone use in the sample of smartphone-based SNS users. Second, similar to existing smartphone research that adapted self-report methods (e.g., Jeong et al., 2020, Kwak et al., 2022), our data may have a reporting bias. Future researchers should overcome this limitation by adopting interview methods or using applications that can trace participants’ smartphone time usage. Third, although we explained the characteristic of anxiety in the context of social experience to explain a predicting role of anxiety in problematic smartphone use, we did not measure a social form of anxiety. It is highly recommended for future researchers to explore more specific forms of anxiety, such as the Fear of Missing Out (FoMO) (Oberst et al., 2017), which is the anxiety of not being able to engage in social networks. Lastly, the generalizability of our results might be limited because the sample for the study only included South Korean. It would be worth exploring further in future studies on samples from different cultural backgrounds, more diversified by age, and considering gender differences.

## 6 Conclusion

Despite its shortcomings, the current study offers insightful suggestions on problematic smartphone use while targeting only smartphone-based SNS users. The results of this study contain some useful indications that would be useful for clinicians working in the field of problematic smartphone use. It would be recommended for future studies to examine more evident cause-effect relationships between various variables and problematic smartphone use, such as smartphone-based SNS users’ need for appealing rewarding experiences through the smartphone. Exploring one’s self-control, anxiety, and motivation level to seek out rewarding experiences would be necessary to suggest intervention and prevention strategies for problematic smartphone use from smartphone-based SNS users. Also, investigating potential protective factors for problematic smartphone use would be needed through further investigation.

**Funding:** This research was supported by the Basic Science Research Program through the National Research Foundation of Korea (NRF) funded by the Ministry of Science, ICT & Future Planning (NRF- 2014M3C7A1062893). The funders had no role in study design, data collection and analysis, decision to publish, or preparation of the manuscript.

**Institutional Review Board Statement:** This study was approved by the Institutional Review Board of Seoul St. Mary’s Hospital, The Catholic University of Korea College of Medicine, Seoul, Korea (IRB number: KC15EISI0103).

**Informed Consent Statement:** Informed consent was obtained from all subjects involved in the study.

**Data Availability Statement:** Survey material is confidential as it may include unanonymized personal information, but minimal data set can be made available upon request. Contact information: Bohyun Jang, Hankook Research Inc.; bhjang@hrc.co.kr.

## CRediT authorship contribution statement

**Min-Jung Kwak:** Conceptualization, Data curation, Formal analysis, Methodology, Writing – original draft, Writing – review & editing. **Dai-Jin Kim:** Funding acquisition, Project administration, Supervision.

## Declaration of Competing Interest

The authors declare that they have no known competing financial interests or personal relationships that could have appeared to influence the work reported in this paper with the National Research Foundation of Korea (NRF) funded by the Ministry of Science, ICT & Future Planning (NRF- 2014M3C7A1062893).

## Data Availability

The data that has been used is confidential.
